# Tick-borne pathogens in *Ixodes ricinus* (Acari: Ixodidae) and *Ixodes inopinatus* × *I. ricinus* hybrids in Central Europe: no link to bird diversity or abundance

**DOI:** 10.1093/jme/tjag046

**Published:** 2026-04-10

**Authors:** Sajjad Ghodrati, Karolína Šimurdová, Alena Hrbatová, Karolina Volfová, Markéta Nováková, Sylvie Ševčíková, Jan Votýpka, David Modrý, Ludek Zurek, Peter Adamík

**Affiliations:** Department of Botany and Zoology, Faculty of Science, Masaryk University, Brno, Czech Republic; Department of Zoology, Faculty of Science, Palacký University, Olomouc, Czech Republic; CEITEC University of Veterinary Sciences, Brno, Czech Republic; CEITEC University of Veterinary Sciences, Brno, Czech Republic; Department of Parasitology, Faculty of Science, Charles University, Prague, Czech Republic; Department of Botany and Zoology, Faculty of Science, Masaryk University, Brno, Czech Republic; Department of Microbiology, Nutrition and Dietetics/CINeZ, Faculty of Agrobiology, Food and Natural Resources, Czech University of Life Sciences Prague, Prague, Czech Republic; Department of Parasitology, Faculty of Science, Charles University, Prague, Czech Republic; Biology Centre, Institute of Parasitology, Czech Academy of Sciences, České Budějovice, Czech Republic; Department of Botany and Zoology, Faculty of Science, Masaryk University, Brno, Czech Republic; Biology Centre, Institute of Parasitology, Czech Academy of Sciences, České Budějovice, Czech Republic; Department of Veterinary Sciences, Faculty of Agrobiology, Food and Natural Resources, Czech University of Life Sciences, Prague, Czech Republic; CEITEC University of Veterinary Sciences, Brno, Czech Republic; Department of Microbiology, Nutrition and Dietetics/CINeZ, Faculty of Agrobiology, Food and Natural Resources, Czech University of Life Sciences Prague, Prague, Czech Republic; Department of Zoology, Faculty of Science, Palacký University, Olomouc, Czech Republic

**Keywords:** birds, *Ixodes inopinatus*, *Ixodes ricinus*, tick-borne pathogens, hybrids

## Abstract

While the presence of *Ixodes inopinatus* in Central Europe has not been conclusively demonstrated, recent studies have provided evidence of *Ixodes ricinus* with introgressed *I. inopinatus* TROSPA alleles in the Czech Republic. Migratory birds have been hypothesized to carry *I. inopinatus* from North Africa to more northern regions, potentially leading to hybridization with *I. ricinus* in Southern and possibly Central Europe. In this study, we screened 659 tick nymphs collected from six sites in the Czech Republic, which were selected based on the diversity and abundance of birds and classified as bird-poor and bird-rich habitats. We did not detect any *I. inopinatus*, but hybrid tick prevalence (individuals bearing different TROSPA alleles) ranged from 0.7% to 5.5% per site and was not associated with habitat type. We found a decline in the occurrence of hybrids along a south-to-north latitudinal gradient. Overall, 22.9% of ticks tested positive for at least one tick-borne pathogen, with a higher prevalence in *I. inopinatus/ricinus* hybrids (30%) than in *I. ricinus* (22.7%). Hybrid ticks harbored *Borrelia burgdorferi* s. l., *B. miyamotoi*, and *Rickettsia helvetica*. In *I. ricinus*, *B. burgdorferi* s. l. was the most prevalent pathogen (11.8%), and by genospecies dominated by *B. afzelii*, followed by *Rickettsia* spp. (5.6%), *Anaplasma phagocytophilum* (4.7%, mostly ecotype I), and *B. miyamotoi* (2.5%), with several co-infection patterns observed. One tick was positive for *Neoehrlichia mikurensis*. The prevalence of *Anaplasma phagocytophilum, Borrelia miyamotoi, Borrelia burgdorferi* s. l., and *Rickettsia* spp. substantially varied among sites and was independent of habitat type.

## Introduction

Ticks are obligate hematophagous arthropods that serve as vectors for numerous pathogens of medical and veterinary importance (Keve et al. 2024). Due to climate change, ticks and tick-borne pathogens are anticipated to undergo a significant northward expansion in their geographical distribution (Rocklov and Dubrow 2020, Estrada-Peña et al. 2021, Ghodrati et al. 2024). In this context, birds (particularly passerines) are likely to play a crucial role in tick dispersal alongside their migratory routes (Hornok et al. 2012, Estrada-Pena et al. 2017, Toma et al. 2021).

*Ixodes ricinus* (Acari: Ixodidae, Linnaeus, 1758) is the dominant tick species in Europe, with a distribution that spans the entire West Palearctic region, extending from the British Isles to the Russian Urals and from North Africa to Scandinavia (Estrada-Pena and de la Fuente 2017, Estrada-Pena et al. 2017). It is a vector of numerous pathogens of veterinary and medical importance, including *Borrelia* spp., *Rickettsia* spp., *Anaplasma phagocytophilum*, *Babesia microti*, and Flaviviruses (Bertola et al. 2021).

In 2014, a new species, *Ixodes inopinatus*, was described (Estrada-Pena et al. 2014) in Spain and later reported in Portugal, Italy, and North Africa (Algeria, Tunisia, Morocco) (Estrada-Pena et al. 2014). *Ixodes inopinatus* has also been reported in several other European countries, including Germany, Austria, Switzerland, Romania, and Turkey, based on morphology and partial 16S rDNA sequencing (Chitimia-Dobler et al. 2017, 2018, Hauck et al. 2019, Bursali et al. 2020, Fares et al. 2021, Kahl et al. 2021, Krol et al. 2022, Mechouk et al. 2022). However, these reports have been questioned in recent studies that analyzed genes other than 16S rDNA (Hrazdilova et al. 2023, Rollins et al. 2023, Danek et al. 2024) and demonstrated that *I. ricinus* is primarily distributed in Europe, what is now called *I. inopinatus* in North Africa, and hybrids of these two closely related species mainly in Southern Europe (Noureddine et al. 2011, Poli et al. 2020, Hrazdilova et al. 2023, Danek et al. 2024). These studies also suggested that migratory birds contributed to the dispersal of *I. inopinatus* into Europe, thereby facilitating hybridization with the local *I. ricinus* population. The Italian Peninsula was identified as a key hybridization zone (Danek et al. 2024).

Furthermore, the most recent publication based on mitochondrial genome sequencing also demonstrated the presence of a separate clade of ticks in North Africa that differed from *I. ricinus* in Europe (Estrada-Peña et al. 2025). Interestingly, sequencing the whole genomes of six ticks from North Africa, identified morphologically as *I. ricinus* and *I. inopinatus*, did not reveal a clear distinction between them (Baede et al. 2024).

The ability of *I. inopinatus* to carry and transmit diseases is not well understood, as most studies have relied on unreliable identification methods (Hauck et al. 2019, del Cerro et al. 2022). Recently, *Borrelia burgdorferi* s.l., *B. lusitaniae*, *Rickettsia* spp., *R. helvetica*, and *R. monacensis* were detected in *I. inopinatus* from Algeria and Italy using molecular methods based on nuclear and mitochondrial genes (Norte et al. 2021, Danek et al. 2024). Hybrids of *I. inopinatus* and *I. ricinus* from Italy were found to carry pathogens typically associated with *I. ricinus* (Danek et al. 2024); however, this finding is based on a limited sample set.

In the current study, we further explored the ecological hypothesis of bird-mediated dispersal of hybrids by analyzing ticks from sites characterized by differences in habitat complexity and bird fauna. We hypothesized that hybrid ticks would be more common in sites with high bird diversity and abundance. We also predicted that the proportion of hybrids would follow the latitudinal gradient observed in Italy: the further north, the lower the proportion of hybrids in the sample (Danek et al. 2024). We should stress that our study does not deal with the taxonomic status of *I. inopinatus.* We investigate the patterns of TROSPA variation consistent with the introgression signals potentially derived from southern tick populations. For the purpose of this study, we consider individual ticks bearing different TROSPA alleles as hybrids. Additionally, we assessed the prevalence of *Anaplasma phagocytophilum*, *Borrelia miyamotoi*, *Borrelia burgdorferi* s.l., and *Rickettsia* spp. in ticks collected from these two types of habitats.

## Materials and Methods

### Bird and Tick Sampling

Based on bird hotspots data from databases such as eBird (https://ebird.org) and Avif (https://avif.birds.cz), we established three study plots in areas with structurally diverse habitats, including alluvial forests, wetlands, and meadows. At each study plot, we established a pair of survey sites: one in a structurally rich habitat (hereafter “rich”) with a high expected bird abundance, and a second site with low structural diversity (hereafter “poor”). The pairs of sites were 1.1, 2, and 1.2 km apart. Prior to plot selection, we inspected the habitat features based on the geospatial data catalog of Ecosystem types of Europe (spatial resolution 100 m, EEA 2015) and aerial photographs.

Bird and tick surveys were conducted sequentially, in the eastern part of Czechia, starting in the southernmost study plot near Valtrovice in the South Moravia region (rich site: 48.78917N, 16.21239E; poor site: 48.79857N, 16.215397E), followed by the more northern study plot in Tovačov-Morávka náhon (rich site: 49.4406067N, 17.2974E; poor site: 49.4448167N, 17.3248E) in the Olomouc region. The northernmost plot in Chomoutov-Březce (Olomouc region) was surveyed last (rich site: 49.6481839N, 17.2421356E; poor site: 49.6578N, 17.23543E).

A standard point-count technique was used to estimate bird abundance and overall species richness (Bibby et al. 2000), a widely applied method for surveying breeding birds. At each sampling point, a single observer recorded all birds heard and seen within a 10-minute interval. Counts at paired sites were always conducted on the same day. The bird count was repeated twice during the breeding season. The first census was performed from late April to mid-May, and the second approximately one month later at the same sites. The censuses were initiated between 5:04 and 6:46 h of CET (mean 6:01 h). All bird census data are available on Zenodo (Ghodrati et al. 2025). Bird abundance is expressed as the mean of the two counts per site, and species diversity as the cumulative number of species detected during both counts per site.

To collect ticks, we conducted two flagging sessions at each site: one in April/early May and another in late May/June 2021 (Supplementary Table S1). All ticks were preserved in 70% ethanol and stored in a freezer prior to processing. We collected 242 larvae, 891 nymphs, and 718 adults. For species identification, we analyzed a subset of 659 nymphs, as this was the only life stage with sufficient numbers from all sites.

### Tick Identification

Each tick was thoroughly washed twice with phosphate-buffered saline (PBS) and homogenized using pestles in a 1.6 ml microtube. The DNA was extracted using a commercial kit (Exgene Cell SV, South Korea) following the manufacturer’s instructions. The DNA was eluted in 100 μl of elution buffer (EB) (Qiagen, Germany) and stored at 4°C until further processing. To identify *I. ricinus, I. inopinatus*, and their hybrids, a multiplex PCR targeting a segment of the *TROSPA* gene, as described by Hrazdilova et al. (2023) and Danek et al. (2024), was conducted. Amplicons were visualized under the UV light, and samples exhibiting an *I. inopinatus* or hybrid pattern, were subjected to an additional PCR targeting a longer fragment (824 bp) of the *TROSPA* gene (Hrazdilova et al. 2023) and sequenced in both directions using the amplification primers through Macrogen sequencing service (Macrogen Europe, The Netherlands).

### Tick-Borne Pathogens

All ticks were screened for *Borrelia burgdorferi* sensu lato, *Borrelia miyamotoi*, *Rickettsia* spp., and *Anaplasma phagocytophilum*. The presence of the pathogens was assessed in DNA isolated from individual ticks by a conventional PCR (*B. burgdorferi* s. l.*, Rickettsia* spp.), qPCR (*B. miyamotoi*), and nested PCR (*A. phagocytophilum*). *B. burgdorferi* s. l. detection was performed by two different conventional PCRs, targeting portions of the 5S-23S (*rrfA–rrlB*) ribosomal RNA intergenic spacer region (IGS), and flagellin B (*FlaB*) encoding gene (Clark et al. 2005, Heylen et al. 2013). For *B. miyamotoi*, the protocol targeting the *glpQ* gene was used, and the positive samples were subjected to a confirmatory nested PCR targeting the same gene (Fomenko et al. 2010, Graham et al. 2016, Janecek et al. 2020). The presence of rickettsial DNA was assessed by PCR targeting a 401-bp fragment of the *gltA* gene to detect the *Rickettsia* genus. Positive ticks were then subjected to a second assay targeting a 632-bp fragment of the *ompA* gene, which is present in Spotted Fever Group (SFG) *Rickettsia* (Regnery et al. 1991, Roux et al. 1996).

The detection of *A. phagocytophilum* was based on an initial nested PCR targeting 407-bp variable segment of the *groEL* gene (Alberti et al. 2005, Jaarsma et al. 2019). Samples successfully amplified in the first nested PCR were then subjected to a second nested PCR targeting a 1,297-bp fragment of the entire *groEL* operon (hereafter referred to as long *groEL*) using slightly modified primers (Liz et al. 2002). To detect the *groEL* ecotype, samples with positive long *groEL* were sequenced. Samples positive for short *groEL* but negative for long *groEL* amplification were sequenced using the short *groEL* amplification primers. The primers and details of all PCR assays are listed in Supplementary Tables S2 and S3. Products of the expected size were either excised from gels and purified using a Gel/PCR DNA Fragments Kit (Geneaid, Taiwan) or cleaned with ExoSAP-IT™ PCR Product Cleanup Reagent (Applied Biosystems™, USA). Purified products were sequenced bidirectionally using the amplification primers. Sequencing was conducted by Macrogen Sanger Sequencing Services, and in the case of *B. burgdorferi* s. l. at the Charles University sequencing facility in BIOCEV.

### Data Analysis

We used the paired-sample Wilcoxon test to compare bird species diversity and overall abundance between poor and rich habitats. Each habitat type was paired within the three plots over the two censuses. As we a priori expected higher bird abundance in rich habitats, we used the one-tailed test. Fisher’s exact test (FET) was applied to compare the prevalence of tick-borne pathogens (TBP) across sites. Confidence intervals for binomial proportions were estimated using the R package *DescTools* (Signorell 2014). All analyses were conducted in R version 4.3.3.

To evaluate the hypothesized latitudinal decline of *I. ricinus/inopinatus* hybrids, we used data from our survey and nymphal tick data extracted from the study of Danek et al. (2024) (Supplementary Table S4). The obtained sequences were analyzed in Geneious Prime^®^ software 2022.0.1 (Kearse et al. 2012) and compared with sequences in the GenBank^®^ dataset using the Basic Local Alignment Search Tool (BLAST). Maximum likelihood (ML) phylogenetic analysis for tick identification and pathogen detection was performed using IQTREE v. 1.6.12 (Nguyen et al. 2015) with 1,000 bootstrap replicates. For tick identification, ML analysis was conducted separately for 16S rDNA, 12S rDNA, and *COI*, using GenBank sequences representing the *R. sanguineus* s.l. complex for all three genes. *R. microplus* (NC_023335) and *R. australis* (NC_023348) were used as outgroups (not shown in the trees).

For the *Anaplasma* phylogeny, *groEL* sequences representing different ecotypes of *A. phagocytophilum* were retrieved from GenBank, and *Anaplasma platys* sequences (AF399916 and AF478129) were used as outgroups (not shown in the trees). Alignments were calculated using the multiple alignment program for amino acid or nucleotide sequences (MAFFT) online version 7.0 (Katoh et al. 2019). The best-fit substitution model was selected based on the Bayesian Information Criterion (BIC) computed by the implemented ModelFinder (Kalyaanamoorthy et al. 2017) for each gene separately. Trees were visualized and edited in FigTree v. 1.4.1.

## Results

### Bird Counts

**Fig. 1.**
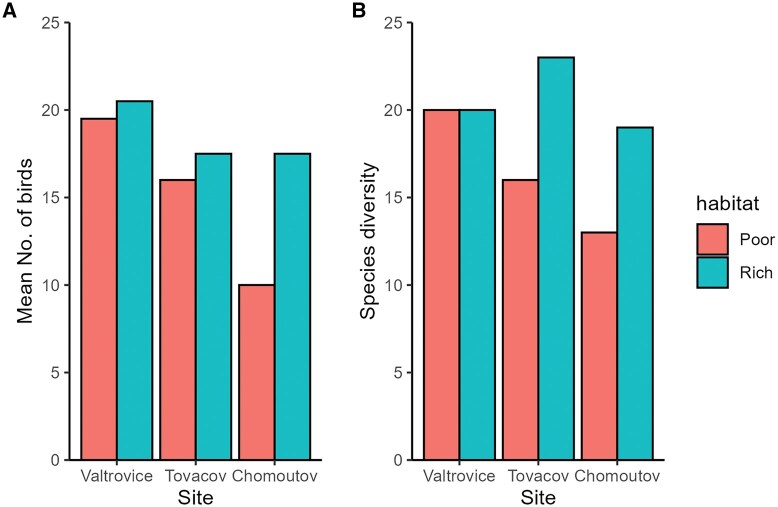
An overview of bird abundance (expressed as the mean number of individuals detected during two-point count sessions) and overall species diversity estimated by point counts at three pairs of study plots. Each plot includes two sites, assigned to either rich or poor habitats.

Overall, across all sites, we detected 42 bird species (Supplementary Table S5). Fewer species were recorded in poor habitats (29) compared to rich habitats (37; Wilcoxon test, *P* = 0.053). Species diversity ranged from as low as 7 per sampling point on a given day to a maximum of 16. Poor habitats had, on average, 11.5 species per census, while rich habitats had 14 species (Supplementary Table S6). Bird abundances per sampling point and day ranged from 10 individuals (poor habitat) to a maximum of 23 (rich habitat). The overall bird abundance was higher in rich habitats (mean ± SE: 18.5 ± 0.8 vs. 15.0 ± 1.9 individuals; Wilcoxon test, *P* = 0.053; Fig. 1, Supplementary Table S6). Species diversity and bird abundances declined from Valtrovice (the southernmost site) towards Chomoutov (the northernmost sites), but only in poor habitats (Fig. 1).

### Ticks

**Fig. 2.**
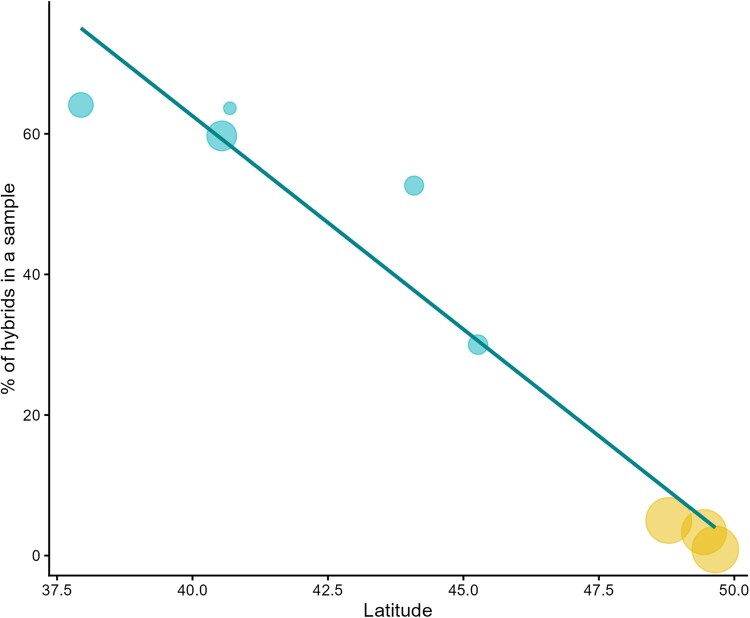
Prevalence of *I. ricinus/inopinatus* hybrids and latitude. Data on tick nymphs were collected from eight field sites in Czechia (yellow) and Italy (blue). The bubble size corresponds to the original sample size. The fitted linear regression line is shown to visualize the pattern. Data source for the five Italian sites: Danek et al. (2024).

Out of the 659 screened ticks, all were classified within the genus *Ixodes*. Twenty ticks (3%) were identified as *I. inopinatus/ricinus* hybrids, while 639 (97%) were identified as *I. ricinus*. No *I. inopinatus* individual was detected. The prevalence of hybrids among all identified ticks ranged from 0.68% to 5.5% per site. However, due to the wide range of 95% confidence intervals (CI), no significant differences were observed between sites (Table 1). Hybrid occurrence was not associated with habitat type (Wilcoxon test, *P* = 0.25; Supplementary Fig. S1). Combined data across all eight sites suggest that the proportion of hybrids decreased with increasing latitude (nymphs only, rho = −0.98, *P* < 0.001; Fig. 2).

**Table 1.** Species diversity of ticks across three pairs of sites in rich and poor habitats (95% confidence intervals for prevalence are in parentheses)

	Poor habitat					Rich habitat				
Site/Tick ID	% hybrids	*n*	% *ricinus*	*n*	Sum	% hybrids	*n*	% *ricinus*	*n*	Sum
**Valtrovice**	5.5 (2–11)	6	94.5 (89–98)	104	110	4.55 (1–10)	5	95.5 (90–99)	105	110
**Tovacov**	3.4 (1–8)	5	96.6 (92–99)	142	147	3.23 (0–11)	2	96.8 (89–100)	60	62
**Chomoutov**	1.2 (0–6)	1	98.8 (94–100)	83	84	0.68 (0–4)	1	99.3 (96–100)	145	146

### Tick-Borne Pathogens

In total, 22.9% (151/659) of ticks tested positive for at least one of the selected TBP. For *I. ricinus*, 22.7% (145/639) of ticks tested positive for at least one TBP. Co-infections were detected in eleven ticks, including three ticks with *R. helvetica + B. afzelii*, two ticks with *A. phagocytophilum* + *B. afzelii*, and one case of each: *A. phagocytophilum* + *R. helvetica*, *A. phagocytophilum* + *B. miyamotoi*, *B. miyamotoi* + *R. monacensis*, *B. miyamotoi* + *B. afzelii*, *B. garinii* + *R. helvetica*, and *A. phagocytophilum* + *R. helvetica* + *B. miyamotoi*.

In *I. ricinus* the most abundant pathogen among the tested ticks was *B. burgdorferi* s. l., with an overall prevalence of 11.8% (74/625), ranging significantly from 1.7% to 22.9% across sites (Pearson’s Chi-squared test, χ^2^ = 19.4, df = 5, *P* = 0.002; Table 2). According to individual genospecies from *B. burgdorferi* s. l. complex, *B. afzelii* was detected most frequently (65/625), followed by *B. garinii* (8/625), and *B. lusitaniae* was detected in only one tick. The overall prevalence of *B. miyamotoi* was 2.5% (16/635), ranging from 0% to 7.7% across sites (Table 2). While significant differences in *B. miyamotoi* prevalence were observed among sites (FET, *P* = 0.023), no clear pattern was detected between habitat types. *Rickettsia* spp. was the second most frequently detected TBP, with an overall prevalence of 5.6% (36/639). Prevalence varied between 4.1% and 8.6% across sites, but the differences were not statistically significant (FET, P = 0.835). *Rickettsia helvetica* was the dominant species (33/639), whereas *R. monacensis* was identified in only three ticks (Table 2).

**Table 2.** Prevalence (%) and sample size (positive/all screened ticks) of tick-borne pathogens (TBP) in *I. ricinus* nymphs across six sites in Czechia

Plot	Valtrovice				Tovačov				Chomoutov			
Habitat	Poor		Rich		Poor		Rich		Poor		Rich	
TBP	%	n/N	%	n/N	%	n/N	%	n/N	%	n/N	%	n/N
***A. phagocytophilum***	8.7	9/104	0.95	1/105	7.7	11/142	0	0/60	0	0/83	6.9	10/145
***Rickettsia* spp.**	4.8	5/104	8.6	9/105	5.6	8/142	5.0	3/60	6.0	5/83	4.1	6/145
***R. helvetica***	3.8	4	8.6	9	4.2	6	5.0	3	6.0	5	4.1	6
***R. monacensis***	1	1	0	0	1.4	2	0	0	0	0	0	0
***B. burgdorferi* s. l.**	14	13/93	22.9	24/105	7.8	11/141	1.7	1/58	16.9	14/83	7.6	11/145
***B. miyamotoi***	7.7	8/104	2.9	3/105	0.7	1/141	1.8	1/57	0	0/83	2.1	3/145

The prevalence of *Anaplasma phagocytophilum* in *I. ricinus* was 4.7% (31/659), ranging from 0% to 8.7% across sites (FET, *P* = 0.002); however, no significant differences were observed between habitat types. We provide a detailed classification of *A. phagocytophilum* based on partial *groEL* gene sequences, following the methodology of previous studies (Jahfari et al. 2014). According to the maximum likelihood (ML) phylogenetic analysis, 22 out of 31 *A. phagocytophilum* sequences (71%) clustered within ecotype I, while the remaining 9 sequences (29%) were assigned to ecotype II. Analysis of *A. phagocytophilum groEL* amplicons also revealed one sample positive for *Neoehrlichia mikurensis*. The sequences showed 100% similarity to complete *N. mikurensis* genomes available in GenBank (accession numbers: CP089285, CP089286, CP066557, CP054597, CP060793). To confirm the BLASTn results, we constructed a maximum-likelihood (ML) phylogenetic tree using IQ-TREE v. 1.6.12 (Nguyen et al. 2015). The sequence clustered closely with other *N. mikurensis* sequences from Europe, supported by a high bootstrap value (Supplementary Fig. S2). Unique representative sequences have been deposited in GenBank under accession numbers: PV067736–PV067748.

For *I. inopinatus/ricinus* hybrids 30% (6/20) of ticks were positive for at least one TBP. One hybrid tick tested positive for *Borrelia miyamotoi* (Valtrovice, poor habitat; prevalence: 5.6%, 1/18), and two hybrids were positive for *Rickettsia helvetica* (Valtrovice, one from rich and one from poor habitat; prevalence: 10.5%, 2/19). *B. burgdorferi* s. l. PCR was overall positive for three hybrids (prevalence 16.7%, 3/18) — namely, *Borrelia valaisiana* detected in one (Tovacov, poor habitat; prevalence 5.6%, 1/18), and *Borrelia afzelii* in two hybrids (Chomoutov, one from rich and one from poor habitat; prevalence 11.1%, 2/18). All hybrid ticks tested negative for *Anaplasma*, and no co-infections were detected.

## Discussion

As expected, we observed higher species diversity and overall abundance of birds in structurally rich habitats. During our survey, we detected only *I. ricinus* and *I. ricinus*/*inopinatus* hybrids. Contrary to our expectation, sites with high avian abundance and diversity were not associated with a greater prevalence of hybrids.

At a broader spatial scale, our results indicate that latitude is a significant predictor of the presence of *I. ricinus*/*inopinatus* hybrids at a given site. In Czechia, the occurrence of these hybrid ticks declines noticeably towards the north. When extending the spatial scale from southern Italy (Danek et al. 2024) to northern Czechia, this trend becomes even more pronounced. These findings support the hypothesis that migratory birds facilitate the northward dispersal of *I. ricinus*/*inopinatus* hybrid ticks from southern Europe (Hrazdilova et al. 2023, Danek et al. 2024). The observed pattern could result from classical dilution by distance: the further away from the source population in Northern Africa, the lower the chance of being dispersed by migratory birds.

The emergence of tick-borne diseases in Europe is shaped by habitat structure and the diversity of vertebrate hosts. Still, it remains underexplored due to the complexity of transmission dynamics and limited large-scale data on key host populations, such as rodents, ungulates, and birds (Ruyts et al. 2018, Takumi et al. 2019). Migratory birds, in particular, present unique challenges for research due to their mobility (Loss et al. 2016, Wilhelmsson et al. 2020, Dumas et al. 2022). Although regional studies exist, the biological relationships between bird species, ticks, and pathogens remain largely unknown, and variations in host diversity significantly impact tick prevalence and pathogen transmission (Schmidt and Ostfeld 2001, Keesing et al. 2010, Hofmeester et al. 2017, Buczek et al. 2020, Keesing and Ostfeld 2021, Dagostin et al. 2024).

In Central Europe, the dominant tick species is *I. ricinus*, which vectors pathogens causing infections such as Lyme borreliosis, tick-borne encephalitis (TBE), anaplasmosis, babesiosis, tick-borne relapsing fever (TBRF), and neoehrlichiosis (Maioli et al. 2012, Portillo et al. 2018, Cunze et al. 2021, Vaclavik et al. 2021). The region’s temperate climate, combined with the expansion of tick habitats driven by changing environmental conditions, has facilitated the spread of these pathogens. In recent years, there has been a notable rise in human cases, attributed to increased awareness, improved diagnostic capabilities, and shifts in tick population dynamics influenced by land-use changes, wildlife population trends, and climate change (Bouchard et al. 2019).

This underscores the need for ongoing surveillance, public health education, and the development of more effective prevention strategies to mitigate the impact of these diseases in Central Europe (Dantas-Torres 2015). Since the evidence of hybridization between *I. ricinus* and *I. inopinatus* was demonstrated (Hrazdilova et al. 2023), several questions remain, including the potential differences in vector competence between *I. ricinus*, *I. inopinatus*, and their hybrids (Danek et al. 2024). In this study, due to the low hybrid abundance (3%), the potential differences in vector competence remain to be clarified. However, among the 20 hybrids, three were positive for *B. burgdorferi* s. l. (one *B. valaisiana* and two *B. afzelii*), two were found to carry *R. helvetica* (10%), and one individual tested positive for *B. miyamotoi*. Based on these findings, *I. ricinus/inopinatus* hybrids might exhibit vector competence similar to that of *I. ricinus*.

The overall prevalence of *B. burgdorferi* s.l. detected in this study was 12%, which corresponds to results from a survey of approximately 11,000 ticks from the South Bohemia region (Hönig et al. 2015). On the contrary, some recent studies from Czechia found a higher prevalence of *B. burgdorferi* s. l. (Richtrová et al. 2022, Balážová et al. 2024). But this is probably because they were conducted in urban areas, where the prevalence of TBPs is generally higher (Rizzoli et al. 2014). Our findings of *B. afzelii* and *B. garinii* as the most prevalent genospecies are also consistent with previous studies from the Czech Republic (Hönig et al. 2015, Richtrová et al. 2022).

The prevalence of *Rickettsia* spp. across bird-rich and bird-poor habitats showed no clear pattern, ranging from 3.8 to 8.6%. This is similar to the 4.8% prevalence of *Rickettsia* spp. in *I. ricinus* nymphs from 201 sites across Czechia (Vaclavik et al. 2021) and the 7.2% prevalence in *I. ricinus* nymphs in Slovakia (Spitalska et al. 2016), with *R. helvetica* also dominant over *R. monacensis* in both studies.

*Anaplasma phagocytophilum* was detected in 4.9% of ticks and was classified into ecotypes I and II. Ecotype I was the dominant ecotype in our study and is known for having the widest host range among all *A. phagocytophilum* ecotypes. These results are consistent with previous findings reported from Czechia (Lesiczka et al. 2023). The distribution of ecotype I is linked to a wide range of mammals, including hedgehogs, red deer, livestock, and humans. In contrast, the distribution of ecotype II is primarily associated with roe deer abundance (Stigum et al. 2019, Grassi et al. 2021).

The site-to-site variability of *B. miyamotoi* may be due to differences in host diversity and abundance. A range of vertebrate hosts has been described, including rodents, deer, birds, raccoons, and wild boars (Cutler et al. 2019); however, no dominant reservoir host has been identified (Gryczynska et al. 2021). In previous studies, no correlation between *B. miyamotoi* infection and geographic region, woodland type, or host-seeking nymph density was observed (Lynn et al. 2018).

Overall, our study provides no evidence for the presence of *I. inopinatus* in Czechia, and confirms a prevalence of *I. ricinus/inopinatus* hybrids comparable to that reported in previous studies (Hrazdilova et al. 2023, Šimurdová et al. 2025). The abundance and species richness of the avian community, together with habitat structural diversity, did not reliably predict the presence of introduced *I. inopinatus* or its hybrids. However, the occurrence of hybrids declined towards northern latitudes, as demonstrated in our previous study (Danek et al. 2024). Based on the available albeit limited data, *I. inopintus* and *I. ricinus/inopinatus* hybrids appear to exhibit vector competence for *Borrelia miyamotoi, B. lusitaniae, Anaplasma* spp., and *Rickettsia* spp. comparable to that of *I. ricinus* (Danek et al. 2024). Norte et al. (2021) studied the population structure of *B. lusitaniae* in North Africa (Algeria) and across Europe, confirming a population division separating samples from southern Portugal and Algeria from those from northern Portugal and other European countries, and suggesting co-evolution between *Ixodes* sp. and *B. lusitaniae*. Clearly, the migration of ticks from Africa to Europe, hybridization and introgression between *I. inopinatus* and *I. ricinus*, and the vector competence of hybrids for tick-borne pathogens require additional studies.

## Supplementary Material

tjag046_Supplementary_Data

## Data Availability

The complete dataset on birds and ticks is available at the Zenodo data repository under the link https://doi.org/10.5281/zenodo.14802255 (Ghodrati et al. 2025).
