# Supplementary material for: Tick-borne pathogens in Ixodes ricinus (Acari: Ixodidae) and Ixodes inopinatus × I. ricinus hybrids in Central Europe: no link to bird diversity or abundance
Source: J Med Entomol. 2026 Apr 10;63(2):tjag046. doi: 10.1093/jme/tjag046 (PMC13070612; doi:10.1093/jme/tjag046)

**Table S1.** An overview of flagging dates (format MDY), timing, and total counts of flagged ticks at each site.

|  |  |  |  |  |  |  |  |
| --- | --- | --- | --- | --- | --- | --- | --- |
| site | plot | date | time from | time until | larvae | nymphs | adults |
| Rich habitat | Chomoutov | 5/17/2021 | 7:50 | 9:14 | 66 | 103 | 131 |
| Rich habitat | Chomoutov | 6/29/2021 | 6:24 | 7:11 | 0 | 43 | 44 |
| Poor habitat | Chomoutov | 5/17/2021 | 10:08 | 11:07 | 2 | 54 | 106 |
| Poor habitat | Chomoutov | 6/29/2021 | 7:35 | 8:23 | 3 | 30 | 70 |
| Rich habitat | Tovačov | 5/12/2021 | 10:26 | 11:34 | 3 | 52 | 51 |
| Rich habitat | Tovačov | 6/28/2021 | 6:58 | 7:52 | 7 | 10 | 12 |
| Poor habitat | Tovačov | 5/12/2021 | 12:15 | 12:58 | 17 | 98 | 20 |
| Poor habitat | Tovačov | 6/28/2021 | 8:33 | 9:25 | 30 | 49 | 14 |
| Rich habitat | Valtrovice | 4/1/2021 | 13:24 | 14:25 | 0 | 144 | 155 |
| Rich habitat | Valtrovice | 5/26/2021 | 12:15 | 13:30 | 46 | 41 | 11 |
| Poor habitat | Valtrovice | 4/1/2021 | 15:20 | 16:20 | 0 | 159 | 66 |
| Poor habitat | Valtrovice | 5/26/2021 | 11:45 | 12:30 | 68 | 108 | 38 |

**Table S2**. Details on primers and all PCR assays.

| **Protocol** | **Targeted species** | **Targeted gene** | **Primer name** | **Primer sequence** | **Product size** | **T_a_** | **Reference** |
| --- | --- | --- | --- | --- | --- | --- | --- |
| **Tick identification** | *Ixodes* spp. | TROSPA-multiplex | Trospa Iric_F1 | GTAAACATCGGCCTAATGG | 362 bp | 52 | Hrazdilova et al., 2023 |
|  |  |  | Trospa Iric_R2 | GGAAAAAATAATGTTAAAACACC |  |  |  |
|  |  |  | Trospa Iino_F2 | GTTGTTCACAGCGAATACT | 233 bp |  |  |
|  |  |  | Trospa Iino_R1 | GAAAAAAATATTAGAACATTAACACTC |  |  |  |
|  |  | TROSPA | Trospa-F2 | TATGGACACGGCGTCGCTGTC | 824 bp | 65 | Noureddine et al., 2011 |
|  |  |  | Trospa-R2 | GCCCAAGCGCATAAATAAGAAGCGG |  |  |  |
| **Pathogens** | *Anaplasma phagocytophilum* | groEL-short product | groEL 1 | ATGGTATGAGTTTGATCGC | 575 bp | 57 | Alberti et al., 2005 |
|  |  |  | groEL 2 | TTGAGTACAGCAACACCACCGGAA |  |  |  |
|  |  |  | groEL 1n | GTGGAATTTGAAAATCCATAC | 407 bp | 55 | Jaarsma et al., 2019 |
|  |  |  | groEL 2n | GTCCTGCTAGCTATGCTTTC |  |  |  |
|  |  | groEL-long product | HS1-modif | TGGGCTGGTARTGAAWT | 1380 bp | 48  52 | Liz et al., 2002 |
|  |  |  | HS6 | CCICCIGGIACIAYACCTTC |  |  |  |
|  |  |  | HS43-modif | ATAGCTAAGGAAGCATAGTC | 1297 bp |  |  |
|  |  |  | HSVR | CTCAACAGCAGCTCTAGTAGC |  |  |  |
|  | *Rickettsia* spp. | gltA | CS-78 F | GCAAGTATCGGTGAGGATGTAAT | 401 bp | 48 | Labruna et al., 2004 |
|  |  |  | CS-323 R | GCTTCCTTAAAATTCAATAAATCAGGAT |  |  |  |
|  |  | ompA | Rr190.70p | ATGGCGAATATTTCTCCAAAA | 632 bp | 58 | Regnery et al., 1991; Roux et al., 1996 |
|  |  |  | Rr190.701 | GTTCCGTTAATGGCAGCATCT |  |  |  |
|  | *Borrelia miyamotoi* | glpQ-qPCR | BmCZglpQ-F | GACCCAGAAATTGACACAACCACAA | 108 bp | 60 | Graham et al., 2016; Janeček et al., 2020 |
|  |  |  | BmCZglpQ-R | TGATTTAAGTTCAGTGAGTGTGAAGTCAGT |  |  |  |
|  |  | glpQ-PCR | Bm OUT fw | CACCATTGATCATAGCTCACAG | 633 bp | 50 | Fomenko et al., 2010 |
|  |  |  | Bm OUT rev | CTGTTGGTGCTTCATTCCAGTC |  |  |  |
|  |  |  | Bm IN fw | GCTAGTGGGTATCTTCCAGAAC | 424 bp | 52 |  |
|  |  |  | Bm IN rev | CTTGTTGTTTATGCCAGAAGGGT |  |  |  |

**Table S3**. *Borrelia burgdorferi* s.l. PCR primers and cycling protocols. The EmeraldAmp GT PCR Master Mix (TaKaRa Bio Inc., Shiga, Japan) was used for *B. burgdorferi* s.l. conventional PCRs according to the manufacturer’s instructions. A total volume of 20 μL was prepared for each reaction containing 10 μL of the Master Mix, 10 pmol of each primer and 1 μL of template DNA, filled with PCR-grade water. The PCR products were visualized using 1% agarose gel electrophoresis under UV light, cleaned with ExoSAP-IT™ PCR Product Cleanup Reagent (Applied Biosystems™, Waltham, MA, USA).

| **IGS conventional PCR** | | |  |
| --- | --- | --- | --- |
| **IGS primers** | ± 390-420bp | Heylen Microbiol 2013, 15:663–673. |  |
| F-primer : | 5'-GAGTTCGCGGGAGAGTAGGTTATTGCC-3' |  |  |
| R-primer: | 5'-TCAGGGTACTTAGATGGTTCACTTCC-3' |  |  |
| **IGS sequencing - short primers** | |  |  |
| F-primer : | 5'-GCGGGAGAGGTTATTGCC-3' |  |  |
| R-primer: | 5'-GTACTTAGATGGTTCACTTCC-3' |  |  |
|  |  |  |  |
| **IGS PCR protocol** | temperature (°C) | Time (hh:mm:ss) | Cycles |
| Taq activation | 94 | 00:15:00 | 1 |
| Denaturation | 94 | 00:00:20 | 10 |
|  |  |  | (lowering 1˚C per cycle) |
| Annealing | 70 | 00:00:30 |  |
| Elongation | 72 | 00:00:30 |  |
| Denaturation | 94 | 00:00:20 | 40 |
| Annealing | 60 | 00:00:30 |  |
| Elongation | 72 | 00:00:30 |  |
| Extension | 72 | 00:07:00 | 1 |
| Hold | 12 | forever |  |
|  |  |  |  |
| **FlaB conventional PCR** | | |  |
| **FlaB primers** | ± 497 bp | Clark *Appl Environ Microbiol*. **2005**, *71:*2616-2625. |  |
| F-primer : | 5'-AARGAATTGGCAGTTCAATC-3' |  |  |
| R-primer: | 5'-GCATTTTCWATTTTAGCAAGTGATG-3' |  |  |
|  |  |  |  |
| **FlaB PCR protocol** | temperature (°C) | Time (hh:mm:ss) | Cycles |
| Taq activation | 96 | 00:05:00 | 1 |
| Denaturation | 95 | 00:00:30 | 30 |
| Annealing | 52 | 00:00:30 |  |
| Elongation | 72 | 00:01:00 |  |
| Extension | 72 | 00:10:00 | 1 |
| Hold | 12 | forever |  |

**Table S4.** Prevalence of *Ixodes inopinatus/ricinus* hybrids across eight sites. For clarity of comparisons, only data on nymphs are presented. (The data source for the Italian sites: Danek et al. 2024).

| site | latitude | hybrids% | n hybrid | n total |
| --- | --- | --- | --- | --- |
| Sicilia | 37.94 | 64.1 | 25 | 39 |
| Basilicata | 40.54 | 59.7 | 40 | 67 |
| Campania | 40.69 | 63.6 | 7 | 11 |
| Emilia Romagna | 44.09 | 52.6 | 10 | 19 |
| Veneto | 45.27 | 30.0 | 6 | 20 |
| Valtrovice | 48.79 | 5.0 | 11 | 220 |
| Tovacov | 49.44 | 3.3 | 7 | 209 |
| Chomoutov | 49.65 | 0.9 | 2 | 230 |

**Table S5.** A full list of detected bird species at rich or poor habitat sites. Migratory status of birds (1 – migratory, 0 – sedentary) is based on whether the central European populations migrate during the non-breeding season to the Mediterranean or to Africa.

| species | rich habitat | poor habitat | migratory status |
| --- | --- | --- | --- |
| Acrocephalus arundinaceus | x |  | 1 |
| Acrocephalus scirpaceus | x |  | 1 |
| Acrocephalus schoenobaenus | x |  | 1 |
| Alauda arvensis |  | x | 1 |
| Alcedo atthis | x |  | 0 |
| Anas platyrhynchos | x |  | 0 |
| Carduelis carduelis |  | x | 0 |
| Certhia familiaris | x | x | 0 |
| Columba oenas |  | x | 1 |
| Columba palumbus | x | x | 1 |
| Corvus corone | x | x | 0 |
| Cuculus canorus | x | x | 1 |
| Cyanistes caeruleus | x | x | 0 |
| Dendrocopos major | x | x | 0 |
| Dryocopus martius | x | x | 0 |
| Emberiza citrinella | x | x | 0 |
| Emberiza schoeniclus | x |  | 1 |
| Erithacus rubecula | x | x | 1 |
| Ficedula albicollis | x | x | 1 |
| Fringilla coelebs | x | x | 1 |
| Gallinula chloropus | x |  | 0 |
| Hippolais icterina | x |  | 1 |
| Luscinia megarhynchos | x | x | 1 |
| Motacila cinerea | x |  | 1 |
| Motacilla alba | x |  | 1 |
| Oriolus oriolus | x | x | 1 |
| Parus major | x | x | 0 |
| Phasianus colchicus | x | x | 0 |
| Phylloscopus collybita | x | x | 1 |
| Pica pica | x | x | 0 |
| Picus viridis | x | x | 0 |
| Poecile palustris | x | x | 0 |
| Prunella modularis | x | x | 1 |
| Remiz pendulinus | x |  | 1 |
| Sitta europaea | x | x | 0 |
| Streptopelia turtur | x |  | 1 |
| Sturnus vulgaris | x |  | 1 |
| Sylvia atricapilla | x | x | 1 |
| Sylvia borin |  | x | 1 |
| Troglodytes troglodytes |  | x | 1 |
| Turdus merula | x | x | 0 |
| Turdus philomelos | x | x | 1 |

**Table S6.** An overview of bird species richness and abundance at each site. Note that each site was survey twice.

| plot | census | site | no. of species | bird abundance |
| --- | --- | --- | --- | --- |
| Chomoutov | first | poor | 7 | 10 |
| Chomoutov | second | poor | 8 | 10 |
| Chomoutov | first | rich | 11 | 17 |
| Chomoutov | second | rich | 15 | 18 |
| Tovačov | first | poor | 13 | 17 |
| Tovačov | second | poor | 14 | 15 |
| Tovačov | first | rich | 13 | 17 |
| Tovačov | second | rich | 16 | 18 |
| Valtrovice | first | poor | 12 | 15 |
| Valtrovice | second | poor | 15 | 23 |
| Valtrovice | first | rich | 15 | 19 |
| Valtrovice | second | rich | 14 | 22 |

**Figure S1.** Relationships between site-related bird species diversity and mean bird abundance with the proportions of detected tick hybrids (n = 6 study sites).


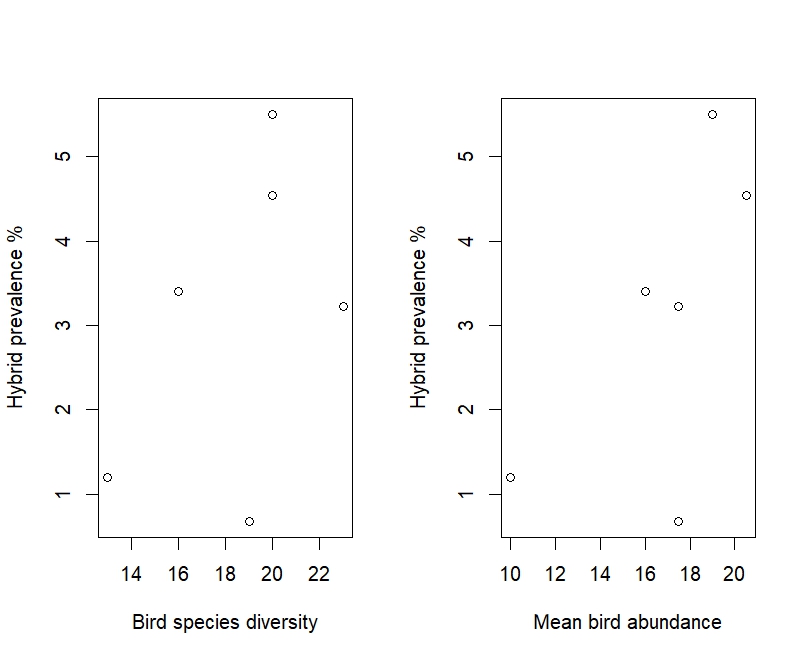


**Figure S2.** The phylogenetic tree of *groEL* was inferred using the maximum likelihood method. Branch support was calculated by 1000 bootstrap replicates. The numbers at the nodes indicate bootstrap values and only values above 50 thresholds are displayed. The sequence from this study (*N. mikurensis* PV067747) is marked in bold in the tree. GeneBank’s acquired sequences are marked by their organism’s name and accession number. Two sequences of *A. phagocytophilum* were used as outgroups. The scale bar indicates the number of substitutions per site.


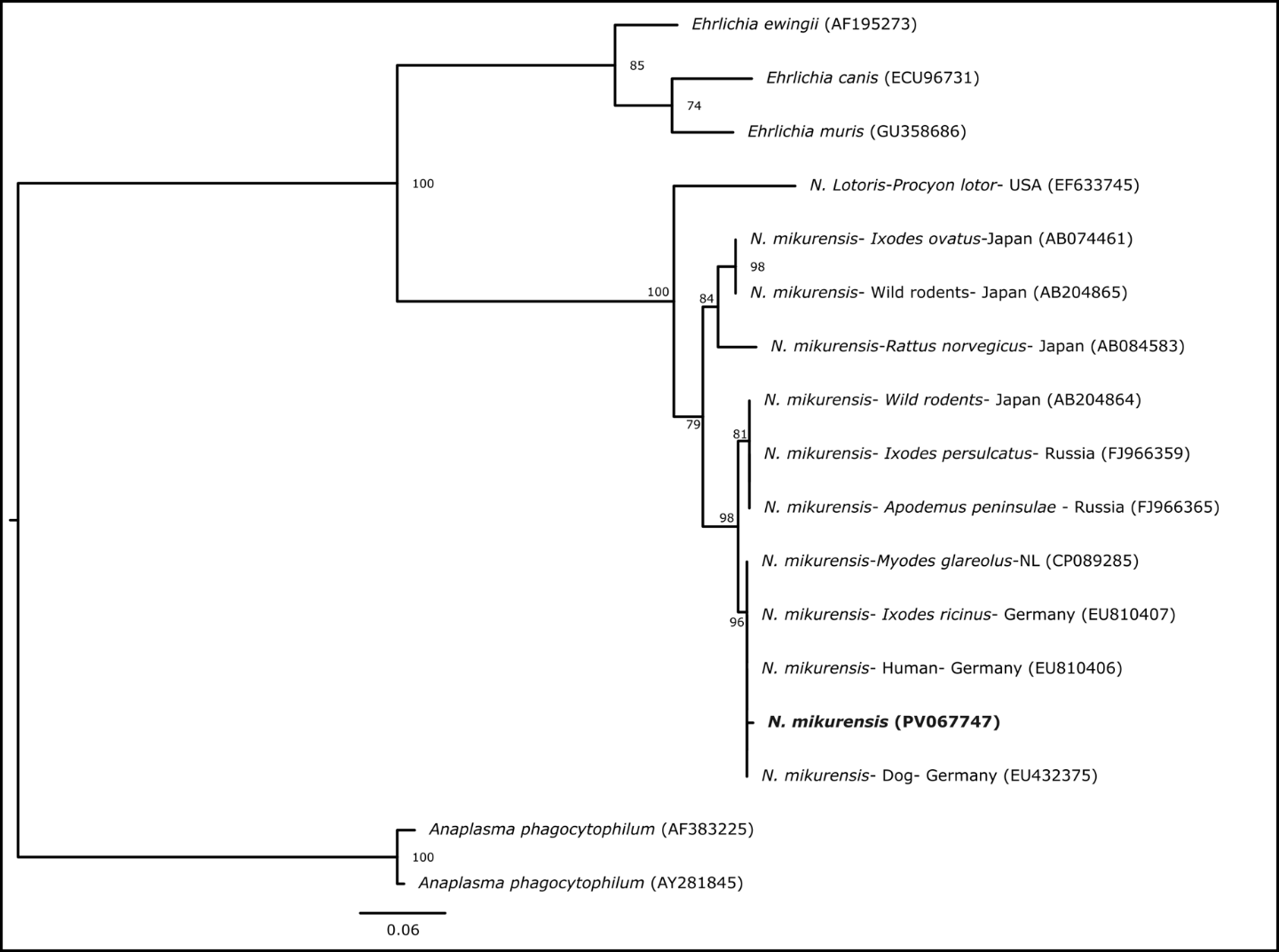

Supplement: tjag046_Supplementary_Data [file tjag046_supplementary_data.docx]
